# Abietic Acid as a Novel Agent against Ocular Biofilms: An In Vitro and Preliminary In Vivo Investigation

**DOI:** 10.3390/ijms25031528

**Published:** 2024-01-26

**Authors:** Monika Dzięgielewska, Marzenna Bartoszewicz, Marta Książczyk, Bartłomiej Dudek, Malwina Brożyna, Patrycja Szymczyk-Ziółkowska, Piotr Gruber, Jacek Pawlak, Weronika Kozłowska, Sylwia Zielińska, Jędrzej Fischer, Aleksandra Woytoń, Adam Junka

**Affiliations:** 1 Eye Institute, Prymasa Augusta Hlonda 10c/u7, 02-972 Warsaw, Poland; 2 Department of Pharmaceutical Microbiology and Parasitology, Medical University of Wroclaw, 50-367 Wroclaw, Poland; marzenna.bartoszewicz@umw.edu.pl; 3 Department of Microbiology, Institute of Genetics and Microbiology, University of Wrocław, 51-148 Wroclaw, Poland; marta.ksiazczyk@uwr.edu.pl; 4 Platform for Unique Model Application, Department of Pharmaceutical Microbiology and Parasitology, Medical University of Wroclaw, 50-367 Wroclaw, Poland; malwina.brozyna@umw.edu.pl (M.B.); aleksandra.woyton@umw.edu.pl (A.W.); 5 Center for Advanced Manufacturing Technologies (CAMT/FPC), Faculty of Mechanical Engineering, Wroclaw University of Science and Technology, Łukasiewicza 5, 50-371 Wroclaw, Poland; patrycja.e.szymczyk@pwr.edu.pl (P.S.-Z.); piotr.gruber@pwr.edu.pl (P.G.); 6 Medical Department, Lazarski University, 02-662 Warsaw, Poland; jacek.pawlak@lazarski.pl; 7 Department of Pharmaceutical Biology and Biotechnology, Division of Pharmaceutical Biotechnology, Wroclaw Medical University, 50-556 Wroclaw, Poland; weronika.kozlowska@umw.edu.pl (W.K.); sylwia.zielinska@umw.edu.pl (S.Z.); 8 Department of Angiology, Hypertension and Diabetology, Wroclaw Medical University, 50-556 Wroclaw, Poland; jedrzej.fischer@umw.edu.pl

**Keywords:** ocular infections, abietic acid, biofilm, bacterial cellulose, *Galleria mellonella*, pathogens

## Abstract

Biofilm-related ocular infections can lead to vision loss and are difficult to treat with antibiotics due to challenges with application and increasing microbial resistance. In turn, the design and testing of new synthetic drugs is a time- and cost-consuming process. Therefore, in this work, for the first time, we assessed the in vitro efficacy of the plant-based abietic acid molecule, both alone and when introduced to a polymeric cellulose carrier, against biofilms formed by *Staphylococcus aureus*, *Pseudomonas aeruginosa*, and *Candida albicans* in standard laboratory settings as well as in a self-designed setting using the topologically challenging surface of the artificial eye. These analyses were performed using the standard microdilution method, the biofilm-oriented antiseptic test (BOAT), a modified disk-diffusion method, and eyeball models. Additionally, we assessed the cytotoxicity of abietic acid against eukaryotic cell lines and its anti-staphylococcal efficacy in an in vivo model using *Galleria mellonella* larvae. We found that abietic acid was more effective against *Staphylococcus* than *Pseudomonas* (from two to four times, depending on the test applied) and that it was generally more effective against the tested bacteria (up to four times) than against the fungus *C. albicans* at concentrations non-cytotoxic to the eukaryotic cell lines and to *G. mellonella* (256 and 512 µg/mL, respectively). In the in vivo infection model, abietic acid effectively prevented the spread of staphylococcus throughout the larvae organisms, decreasing their lethality by up to 50%. These initial results obtained indicate promising features of abietic acid, which may potentially be applied to treat ocular infections caused by pathogenic biofilms, with higher efficiency manifested against bacterial than fungal biofilms.

## 1. Introduction

Eye infections are among the most prevalent conditions in ophthalmology. Due to its location and physiology, the eye is highly exposed and susceptible to colonization by microorganisms. If colonization develops into infection, it can lead to significant vision impairment or even total vision loss. Conjunctivitis stands as the primary reason for visits to ophthalmology clinics, with a significant percentage (65–78%) of bacterial conjunctivitis cases affecting children. Symptoms of this infection include redness, pain, burning sensation, abnormal discharge in the conjunctival sac, conjunctival swelling, and eyelid edema [1,2]. In turn, perioperative and postoperative infections in ophthalmic surgeries occur in roughly 1 out of every 1500 patients subjected to an ophthalmic procedure [3]. Postoperative complications, such as necrotizing scleritis and endophthalmitis (an inflammation of the inner eye), can also lead to vision loss.

There have been observations of bacterial biofilms during ophthalmic surgeries, notably during cataract surgeries with intraocular lens (IOL) implantation, as well as after the use of intradural and intraocular implants. Multiple studies have also reported the presence of microbial biofilms on the eye’s conjunctival surface [4,5,6,7,8,9]. Biofilms in the context of eye infections are adherent microbial communities embedded within a protective extracellular matrix, showcasing heightened resilience against antimicrobials. The persistence of these biofilms has driven the search for innovative therapeutic strategies aimed at both their prevention and eradication.

Distinct microbial species are causative agents of biofilm-related infections. Conjunctivitis, and particularly postoperative inflammation, is majorly attributed to Gram-positive bacteria like staphylococci (*Staphylococcus aureus*, *Staphylococcus epidermidis*) and streptococci (*Streptococcus pyogenes*). Other less prevalent causative agents include *Hemophilus influenzae*, *Moraxella lacunata*, *Chlamydia trachomatis*, *Pseudomonas aeruginosa*, and *Candida albicans*. Of these, methicillin-resistant *Staphylococcus aureus* (MRSA) is the predominant pathogenic bacterium, responsible for purulent conjunctivitis and infections of the eyelids’ skin and tissues, especially in patients undergoing ophthalmic surgeries [10,11,12,13,14,15,16,17,18,19,20]. The resistance of bacteria to antibiotics complicates the treatment [21,22]. Ophthalmic infections can originate from bacteria on the eye’s surface, the eyelids, or from items like contact lenses, which can allow the bacteria to penetrate deeper eye layers. Bacterial keratitis linked with contact lenses is predominantly caused by Gram-positive bacteria (mainly *Staphylococcus* spp.), followed by Gram-negative ones like *P. aeruginosa* [23]. Notably, *P. aeruginosa* is not naturally present in the eye. Contact lenses provide an apt environment for these microbes to grow, form resilient biofilms, and initiate infections. Additionally, the acrylic material in IOLs is susceptible to biofilm formation, particularly by Gram-positive pathogens [24,25]. Among fungal strains, *C. albicans* species is a significant causative agent for eye infections, and its treatment becomes difficult due to the biofilm-forming ability and other associated issues, such as reduced drug efficacy and increasing resistance to antifungal compounds. For bacterial eye infections, bactericidal antibiotics with broad-spectrum activity against both Gram-positive and Gram-negative bacteria are recommended. Preference is generally given to drugs like aminoglycosides and fluoroquinolones over bacteriostatic alternatives; in turn, fungal infections need application of such antimicrobial agents as natamycin, voriconazole, or fluconazole [10].

The search for non-antibiotic antimicrobial agents is a crucial need due to the increasing rate of bacterial and fungal resistance against currently used drugs. Abietic acid (ABA), a primary constituent of the pine resin called Resina Pini, has demonstrated multiple beneficial effects, including antimicrobial properties. Though traditionally used in Korean and Japanese medicine, modern studies have highlighted its effectiveness against bacterial strains, and when combined with antibiotics, even against resistant ones [26,27].

Optimizing the therapeutic effects of local ophthalmic drugs can be achieved also by formulating suitable carriers that deliver the drug directly to the target site. Bacterial cellulose (BC) polymer, produced by the *Komagataeibacter xylinus* bacteria, stands out in this context due to its biocompatibility, mechanical strength, and ease of sterilization. Its high purity compared to plant-derived cellulose makes it especially suited for precise applications, like in synthetic corneas [28].

This research, therefore, aims to investigate the efficacy of ABA alone and ABA in BC carriers against bacterial and fungal eye infections, potentially offering a localized treatment solution against infections caused by Gram-positive and Gram-negative bacteria and fungi.

## 2. Results

The objective of the study was to evaluate both the action of ABA against microbial growth and its role in destroying or reducing preformed biofilm. The evaluation of ABA’s prophylactic antimicrobial activity was not considered, because this in vitro study aimed to demonstrate ABA’s potential in combating existing eye infections, rather than maintaining the eye in a sterile state. In the initial phase of the study, the minimum inhibitory concentration (MIC) and minimum bactericidal/fungicidal concentration (MBC/MFC) of ABA were evaluated using a conventional 96-well microtiter assay. The data revealed that ABA possesses antimicrobial efficacy against three standard pharmacopeial microorganisms: *S. aureus*, which has a Gram-positive cell wall structure; *P. aeruginosa*, characterized by a Gram-negative cell wall; and the fungal organism *C. albicans*, whose cell wall is primarily composed of glucan and chitin (see Table 1). The ABA compound demonstrated bactericidal and fungicidal properties against the tested pathogens, with the most pronounced effectiveness observed against the Gram-positive cocci. In contrast, it exhibited the least potency against the yeast-like fungus *C. albicans.*

**Figure 1:**
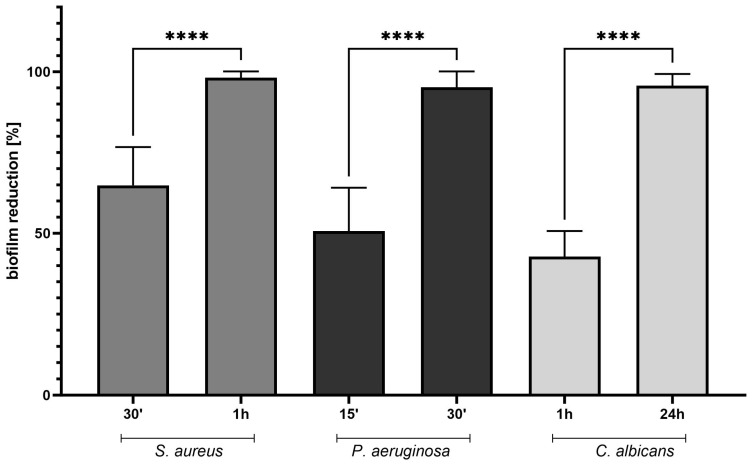
Results of quantitative biofilm oriented antiseptic test (BOAT) performed to assess activity of ABA against microbial biofilm within respective contact times of 30 min and 1 h. ABA—abietic acid. Asterisks indicate the level of statistical significance.

The conducted biofilm analyses utilizing a 96-well microtiter platform (refer to Figure 1) showed that ABA manifested considerably reduced antibiofilm efficacy contrasted with its efficacy against planktonic cells of *S. aureus*, *P. aeruginosa*, or *C. albicans* (compare Table 1 to Table 2). Within the tested concentration range of ABA, administering 512 µg/mL resulted in an approximate 40% (*P. aeruginosa*) to 70% (*S. aureus*) reduction of bacterial biofilm formation and around a 20% reduction in fungal biofilm development. At the maximal concentration examined, specifically 1024 µg/mL, the biofilm reduction rates for *S. aureus*, *P. aeruginosa*, and *C. albicans* were approximately 80%, 70%, and 50%, respectively. In the assessment of biofilm reduction, concentrations of 256 µg/mL and 512 µg/mL did not differ markedly (ca. 10%) across all biofilms tested within a 24-h contact period. Given the particular focus of this research on the potential use of ABA as a treatment for ocular infections, subsequent testing was conducted using the biofilm oriented antiseptic test (BOAT) at clinically relevant exposure times of 15 and 30 min, as well as 1 and 24 h, and ABA concentration equal to 256 µg/mL to establish a control benchmark (see Table 3).

The outcomes of the qualitative BOAT test are binary, providing an assessment of which specific contact times and concentrations of ABA lead to a significant reduction in microbial biofilm to levels undetectable by the employed viability assays, analogous to the procedures used in MIC determinations. The findings detailed in Table 3 demonstrate that ABA exhibits a comparable degree of efficacy at contact times of 15 and 30 min for bacterial biofilms. However, its effectiveness against fungal biofilms is notably reduced when contrasted with the proven activity of polyhexamethylene biguanide (PHMB) that is clinically applied antiseptic agent, herein used for the same pathogens the ABA was used against.

For a more comprehensive understanding of ABA’s effectiveness within clinically pertinent exposure times of 30 min and 1 h, a quantitative BOAT test was also conducted (as shown in Figure 1).

The quantitative BOAT demonstrated that ABA exerted greater antimicrobial effects (within reduced exposure durations) against *P. aeruginosa* as opposed to *S. aureus*. However, its efficacy was moderate against *C. albicans*. Notably, a 1 h exposure achieved roughly a 40% reduction in fungal biofilm, whereas exposure for merely 30 min was sufficient to attain approximately 60% and 90% reductions in staphylococcal and pseudomonal biofilms, respectively.

**Figure 2:**
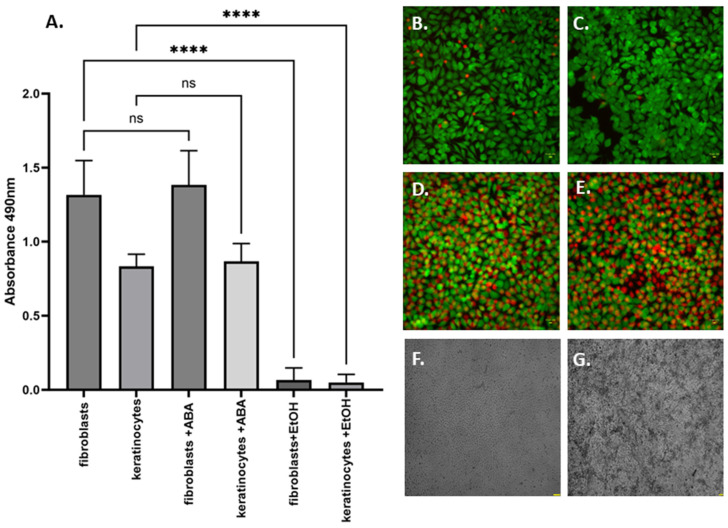
(**A**) Cell viability of fibroblasts and keratinocytes with abietic acid treatment. The leftmost two bars display the baseline viability of fibroblast and keratinocyte cell lines, respectively. The descriptor “+ABA” indicates the viability of these cell lines post-treatment with ABA. For reference, 70% ethanol (EtOH), a compound with established cytotoxicity, was utilized as a control. Significance levels between groups are denoted by asterisks: “****” denotes high significance level of 0.0001; “ns”–lack of statistical significance as determined by ANOVA with Tukey’s multiple comparison test. (**B**,**C**) Representative pictures of fibroblasts and keratinocytes, respectively, treated with ABA. (**D**,**E**) Fibroblasts and keratinocytes, respectively, treated with 70% EtOH. The green-dyed shapes are cells of intact cell membrane (“live cells”), while red-dyed cells are of damaged cell membrane (“dead cells”). The black areas are surface devoid of fluorescent cells. (**F**,**G**) Images of fibroblasts and keratinocytes, respectively, made with bright field microscopy. LumaScope 620, magn. 20×, SYTO-9/Propidium Iodide dye; yellow bar denotes length of 10 µm.

Upon establishing the concentrations and exposure times required to substantially diminish the biofilms under investigation, an in vitro cytotoxicity assessment of 256 µg/mL of ABA on eukaryotic cells, specifically fibroblasts and keratinocytes, was conducted. The data illustrated in Figure 2 suggest that the viability of these cell lines remained unaffected following exposure to ABA. In contrast, the introduction of the control substance, 70% ethanol, resulted in a significant decrease in fibroblast and keratinocyte viability, thus confirming the validity of the experimental model employed.

**Figure 3:**
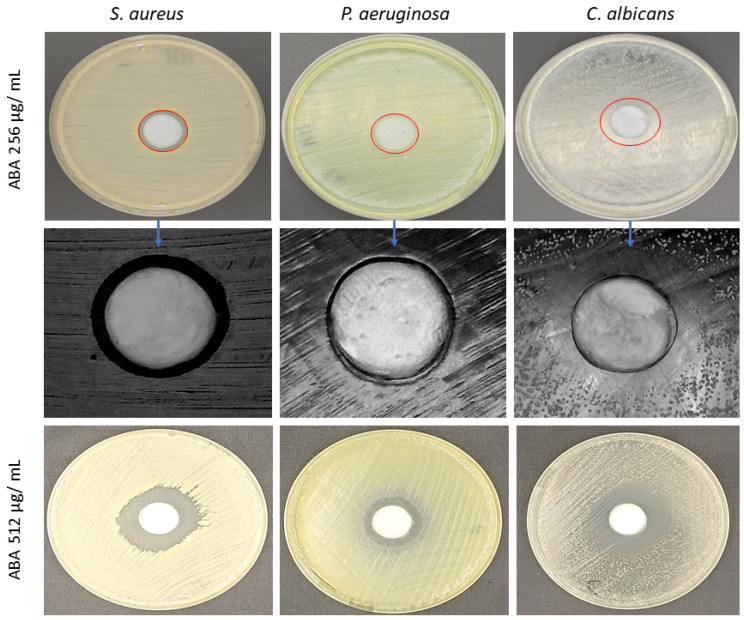
Zones of inhibition induced by ABA released from BC. The delineated red circles differentiate the clear zone of inhibition from the surrounding area where a reduced density of colony growth is present, albeit not completely absent. Blue arrows indicate magnification of inhibition zones resulting from release of 256 µg/mL ABA.

Subsequently, the optimally determined concentrations of ABA were incorporated into a biocellulose (BC) carrier and applied to agar plates inoculated with *S. aureus*, *P. aeruginosa*, and *C. albicans*, following a procedure analogous to the antibiotic disk diffusion method (refer to Figure 3). The resultant zones of inhibition corroborated the capacity of ABA to be effectively released from the BC carrier, as well as the antimicrobial properties of ABA, which were consistent with the findings obtained from alternative research methodologies previously utilized.

The quantitative findings from this research are delineated in Table 4. While 256 µg/mL ABA demonstrated antimicrobial efficacy against all the tested pathogens, *P. aeruginosa* exhibited the greatest tolerance to the released ABA. Conversely, the inhibition zones for *S. aureus* and *C. albicans* were quite comparable, with diameters measuring approximately 20–24 mm on average.

**Figure 4:**
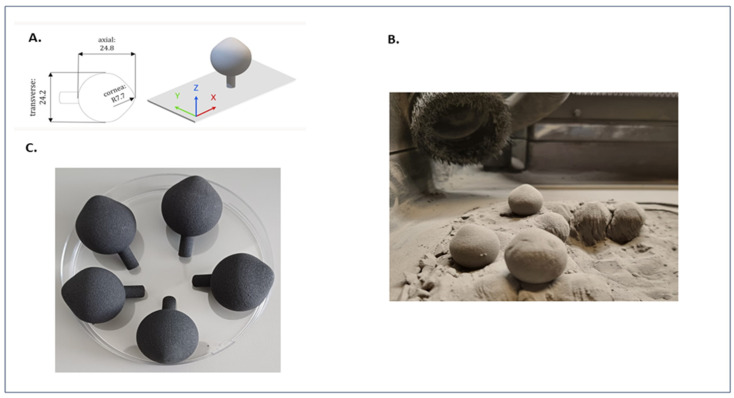
Production of artificial eyeball. (**A**) 3D project. (**B**) Additive manufacturing. (**C**) Ready-to-use product.

To simulate the complex topography of the human eyeball, artificial eyeballs were created utilizing 3D modeling techniques and produced through additive manufacturing technology (Figure 4).

**Figure 5:**
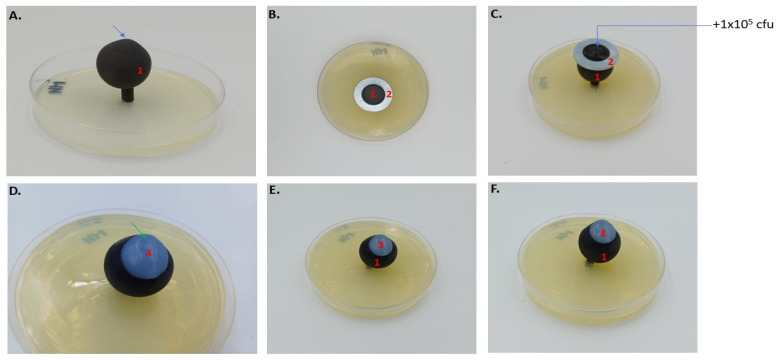
Experimental arrangement for testing ABA-BC carrier on an artificial eyeball. Panel (**A**) illustrates the artificial eyeball (1) with a naturally irregular shape (blue arrow highlighting the asymmetry on the eye’s surface) embedded in an agar plate. The setup includes a stabilizer (2), shown in Panels (**B**,**C**), which prevents microbial suspension from dispersing. Panel (**D**) displays the placement of the ABA-BC carrier (3) on the contaminated surface of the artificial eyeball for the duration of the contact time. Panels (**E**,**F**), captured from different perspectives, demonstrate the ABA-BC carrier’s adherence (green arrow) to the irregular topography of the artificial eyeball model.

**Figure 6:**
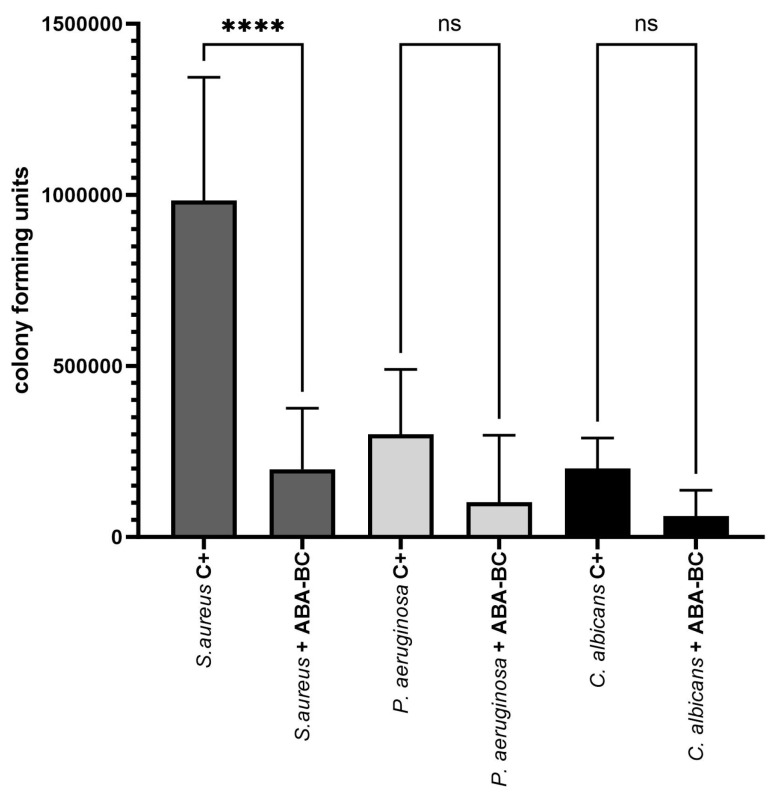
The number of cells that survived the exposure to ABA-BC compared to non-treated control setting (C+) in the model of artificial eye. Significance levels between groups are denoted by asterisks: “****” denotes high significance level of 0.0001; “ns”–lack of statistical significance as determined by ANOVA with Tukey’s multiple comparison test.

Subsequently, the artificial eyeballs were employed in a novel in vitro model designed to evaluate the antimicrobial efficacy of the ABA-BC carrier against *S. aureus*, *P. aeruginosa*, and *C. albicans* (Figure 5 and Figure 6).

**Figure 7:**
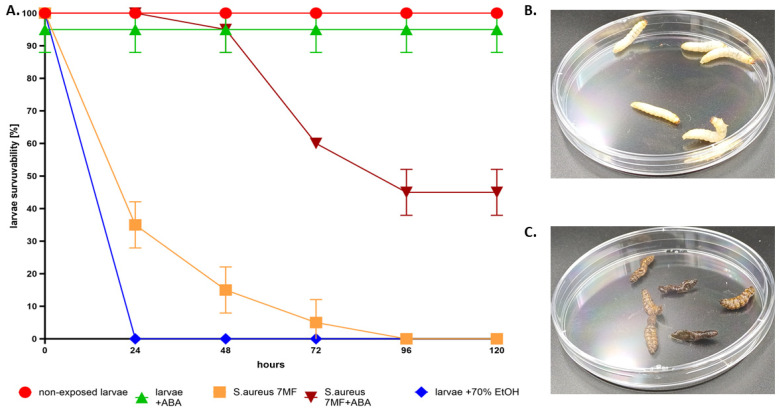
(**A**) The survivability (%) of larvae in the following 0–120 h after exposure to 256 µg/mL of ABA compound, or 70% ethanol, or the DMSO-containing PBS buffer (non-exposed larvae, control of growth). The infection model consisted of larvae injected with 7 McFarland of *S. aureus* together with 256 µg of ABA compound. In total, 100 larvae were used for this experiment. (**B**) Live, creamy, motile larvae not exposed to any treatment. (**C**) Dead, flaccid, motionless larvae after exposure to EtOH.

The results from this quantitative analysis, as depicted in Figure 6, suggest that the application of the AA-BC carrier within an exposure duration that is non-cytotoxic (referencing Figure 3) results in a substantial and significant reduction of microbial biofilm on the surface of the artificial eyeballs. Finally, the preliminary in vivo evaluation was conducted using the *Galleria mellonella* larva model to concurrently assess the cytotoxicity and antimicrobial efficacy of ABA. For this aspect of the study, the *S. aureus* strain was selected for investigation. The outcomes of this analysis are illustrated in Figure 7.

The data illustrated in Figure 7 demonstrate that the introduction of ABA into a live organism—serving as a model for human tissue in this study due to the presence of differentiated cell lines and tissue-like conditions—significantly protects it from the proliferation of an infection caused by *S. aureus*. In the absence of treatment, the infection correlated with a 60% mortality rate within 24 h of microbial inoculation, escalating to a 95% mortality rate within 72 h post-infection. 

## 3. Discussion

The formation of bacterial and fungal biofilms significantly enhances resistance to antibiotics and antifungals, posing a formidable challenge in the management of persistent diseases, including ocular infections [1]. This issue has become a global concern, spurring extensive research into novel approaches for tackling these resilient microbial communities. The approach taken in this study is an exploration of plant-derived bioactive metabolites known for their anti-biofilm properties. Recent studies have shed light on the potential of natural compounds, including phenols, essential oils, terpenoids, lectins, and alkaloids. These compounds demonstrate not only the ability to hinder the formation of biofilms but also possess the capability to disrupt and dismantle established biofilm structures [2,3]. Unlike conventional antibiotics that target specific bacterial processes or structures, plant-derived compounds tend to exhibit a broader spectrum of activity, disrupting biofilm integrity and resilience through multiple pathways [29]. Therefore, our study delves into the antimicrobial potential of plant-derived metabolite, referred to as abietic acid (ABA), against biofilms formed by Gram-positive *S. aureus*, Gram-negative *P. aeruginosa*, and *C. albicans* fungus. To deliver ABA to the infection site we have proposed another nature-derived compound, namely bacterial cellulose carrier.

In our initial assays using a 96-well microtiter plate, the minimum inhibitory concentration (MIC) and minimum bactericidal/fungicidal concentration (MBC/MFC) values indicated that ABA was more potent against planktonic forms of *S. aureus* and *P. aeruginosa* compared to those of *C. albicans* (Table 1). Notably, at an ABA concentration of 512 µg/mL, there was a 50% reduction in bacterial biofilm but only 20% reduction in fungal biofilm (Table 2). Increasing the concentration of ABA to 1024 µg/mL provided a marginal advantage in inhibiting *S. aureus* biofilms over *P. aeruginosa* biofilms, with a 10% differential in efficacy. However, this concentration was more effective against *C. albicans* biofilm, achieving a 40% reduction (Table 2). In assessing the efficacy of ABA through the qualitative biofilm oriented activity test (BOAT), we observed a noteworthy pattern in biofilm reduction over time (Table 3). For bacterial biofilms, a significant decrease was evident within an average of 15 to 30 min. In contrast, a substantial reduction in fungal biofilms was observed only after 24 h. This disparity in response times once again underscores the differential susceptibility of bacterial and fungal biofilms to ABA, suggesting a need for varied therapeutic strategies.

For a comparative analysis, polyhexamethylene biguanide (PHMB), a clinically used antiseptic agent, was included in our tests. In vitro studies, such as those conducted by Kamaruzzaman et al. highlighted that PHMB can eliminate 99.9% of *S. aureus* bacteria, outperforming agents like enrofloxacin, and can reduce biofilm biomass by 28–37% [22]. However, it is notable that PHMB concentrations ranging from 0.005% to 1.0% (*v*/*v*) have shown a certain level of cytotoxicity against cultured human keratinocytes and mouse fibroblasts over a 24–72 h incubation period [23]. In the context of ophthalmology, PHMB’s application is particularly relevant. While higher concentrations exhibit cytotoxic effects, much lower concentrations (around 0.0005%) are prevalent in contact lens care solutions and are a common ingredient in moisturizing eye drops.

At the same time, ABA and its derivatives are increasingly recognized for their antibacterial activity. A study by Helfenstein et al. in 2017 highlighted the potency of AA, reporting a minimum inhibitory concentration (MIC) of 60 μg/mL against methicillin-sensitive *S. aureus* and an even lower MIC of 8 μg/mL against methicillin-resistant *S. aureus*, *S. epidermidis*, and *Streptococcus mitis* [4]. Another investigation noted an MIC value of 800 μg/mL against *S. epidermidis*, illustrating the variability in ABA’s effectiveness against different bacterial strains.

In a subsequent phase of our study, using the quantitative BOAT, ABA demonstrated varied antimicrobial capabilities. When exposed for a shortened duration of 30 min, ABA showed a higher effectiveness against *P. aeruginosa* biofilms, with a 90% reduction, compared to a 60% reduction in *S. aureus* biofilms (Figure 1). This differential response suggests a more potent action of ABA against Gram-negative bacteria under these conditions.

To advance both in vitro and in vivo studies, it was imperative to assess the cytotoxicity of abietic acid (ABA) and establish a concentration threshold that would not induce cellular damage. The preservation of the cell membrane integrity in fibroblasts and keratinocytes at an ABA concentration of 256 µg/mL indicated its non-cytotoxic nature (Figure 2). This finding paved the way also for in vivo experiments using *Galleria mellonella* larvae, chosen due to their diverse cell lines and tissue-like conditions, facilitating an objective assessment of organism survival under experimental conditions.

In BC-using experimental setup (Figure 3, Table 4), the efficacy of ABA against *P. aeruginosa* was notably lower, compared to results presented in Figure 3. In contrast, the growth inhibition zones for both *S. aureus* and *C. albicans* were similar and more pronounced. These observed discrepancies in ABA’s antibacterial action, particularly against Gram-negative bacteria in the microtiter test and on agar media, could be attributed to several factors. These include the nature of the substrate used, the environmental conditions of the study, the distinct cell wall structures of Gram-negative bacteria compared to Gram-positive bacteria, their adhesion capabilities, the rate of bacterial cell division within biofilms, inter-bacterial communication, and the enhanced activity of proton pumps involved in antibiotic resistance [29].

Maria Gabriely de Lima Silva et al. [19] highlighted the inhibitory potential of ABA against both Gram-positive and Gram-negative bacteria. When used in conjunction with antibiotics, ABA demonstrated a marked synergistic effect, particularly against multidrug-resistant strains. This raises two notable hypotheses: firstly, ABA might be disrupting the efflux pump mechanism, a critical component in bacterial drug resistance. Secondly, the synergistic impact against multidrug-resistant bacteria could stem from ABA’s interaction with the bacterial cell wall/membrane, potentially inducing alterations in their permeability. In turn, as outlined by de Silva et al. [20], the mechanisms of ABA’s action on fungal cells are multifaceted, including disrupting the fungal cell membrane, interfering with cell signaling pathways, or affecting enzymes critical for cell wall synthesis.

Another innovative aspect of our study involved the use of an ABA-CB (abietic acid-cellulose biofilm) carrier placed on an artificial eyeball created using 3D modeling techniques (Figure 5 and Figure 6). A study by Samimi et al. in 2013 highlighted the diversity of microorganisms, particularly fungal pathogens like *C. albicans* and Gram-negative bacteria such as *P. aeruginosa*, cultured from orbital implants. Moreover, scanning electron microscopy of selected samples revealed the presence of mixed-species biofilms on porous polyethylene orbital implants [24]. In our study, the ability of ABA-BC to reduce pathogenic biofilm from uneven surfaces of artificial eyeball was proven, with the highest significant efficiency observed towards *S. aureus* (80%) and the lowest against *C. albicans* (69%) (Figure 6).

In the in vivo tests, the injection of ABA into the larvae resulted in their survival with no tissue damage observed over a 120 h observation period. When ABA was administered alongside *S. aureus*, 50% of the larval population survived, whereas larvae infected solely with *S. aureus* survived only up to 72 h. In contrast, the control group exposed to 70% ethanol, exhibited 100% mortality within 24 h (Figure 7) These findings substantiate the bactericidal efficacy of ABA against *S. aureus* in a living organism, marking a pioneering achievement in this field.

The determination of ABA’s non-cytotoxic concentration holds promising implications for its future application in locally administered antibacterial preparations, particularly in ophthalmology. The challenge of antibiotic resistance in ocular infections necessitates solutions to the inherent limitations in the local administration of ophthalmic drugs, particularly those affecting bioavailability [30]. It should be emphasized that the potential application of ABA at a concentration of 256 µg/mL represents an advantage compared to numerous antiseptic agents (which may be applied in such concentrations as 1000 µg/mL), as it is effective against microbes yet displays a non-cytotoxic effect, while harmful effects towards eukaryotic cells are a common disadvantage of many antiseptics currently in use [31]. The effective concentration of ABA positions this molecule as a candidate for topical application, specifically as an antiseptic agent. However, it should be noted that no known microbial resistance mechanisms against ABA have been identified. Thus, this compound may be considered not only an attractive alternative but also a potential complement to antibiotic treatments in combating the threat posed by the spread of antibiotic-resistant microorganisms.

Although the cohesive spectrum of techniques was applied in this research, we are also aware of some disadvantages of our work. Our study focused on reference strains of *S. aureus*, *P. aeruginosa*, and *C. albicans*. However, considering the evolving nature of clinical strains, further research encompassing a broader spectrum of clinical isolates is recommended to fully evaluate the scope and efficacy of ABA in various infectious scenarios. Also, valuable data would be provided if a study on ABA-BC activity were performed on the mammalian models. Nevertheless, by provision of the results presented in this manuscript, the promising alternative for using antiseptics and antibiotics in treatment of eye infections is opened and can be further developed in subsequent research.

## 4. Materials and Methods

### 4.1. Strains and Antimicrobials

The following bacterial and fungal reference strains from American Type and Cell Collection (ATCC, Manassas, VA, USA) were applied for experimental purposes: methicillin resistant *Staphylococcus aureus* (MRSA) 33591, *Pseudomonas aeruginosa* 27853, *Komagataeibacter xylinus* 10245, and *Candida albicans* 10231.

The abietic acid (ABA, Sigma-Aldrich, St. Louis, MO, USA) was dissolved using dimethyl sulfoxide (DMSO, POCH, Lublin, Poland) to stock concentration (25 mg/mL). The polihexanide-containing antiseptic agent (Prontosan, later referred to as the “PHMB”, B. Braun, Meslungen, Germany) was used as comparator to ABA.

### 4.2. Determination of Minimal Inhibitory Concentration and Minimal Bactericidal/Fungicidal Concentration of ABA Using Microtiter Plate Method

The bacterial and fungal strains were cultured overnight in Tryptic Soy Broth (TSB, Biomaxima, Lublin, Poland). Next, 100 µL of sterile TSB was introduced to the wells of the 96-well plate (Sigma-Aldrich, St. Louis, MO, USA) and then 100 µL of ABA solution was introduced using serial dilution in a manner that the highest ABA concentration was 1024 µg/mL (in 100 µL), while the lowest one was 1 µg/mL.

The 0.5/0.8 MacFarland standard of bacterial/fungal suspensions was obtained in a TSB medium using a densitometer (Densilameter II Erba Lachema, Brno, Czech Republic) and diluted 1000× in TSB. Next, 100 µL of microbial solution was introduced into the wells containing ABA solutions. Controls for microbial growth (suspensions in TSB without antimicrobial) and medium sterility (TSB alone) were set up. The experiment’s usability control was microbial solutions subjected to the PHMB activity. The whole setting was incubated at 37 °C/24 h. After incubation, 20 µL of 1% (*w*/*v*) TTC (2,3,5-triphenyltetrazolium chloride, AppliChem Gmbh, Darmstadt, Germany) in TSB was introduced into wells, followed by a 2 h incubation at 37 °C. The first colorless well neighboring red-colored well (resulting from microbial metabolic activity, colorless TTC changes into red formazan) was considered the well containing MIC of ABA. Parallel, from such a well (non-treated with TTC), whole volume of suspension was taken and introduced into 49.9 mL of sterile TSB in a falcon-type tube (Sigma-Aldrich, St. Louis, MO, USA). The suspensions in falcon tubes were incubated for 72 h at 37 °C. After that time, if no increase of turbidity level was observed, the concentration of ABA applied in this setting was considered minimal bactericidal/fungicidal concentration. The measurements were performed in three independent, time-divided experiments.

### 4.3. Determination of Minimal Biofilm Eradication Concentration Using Microtiter Plate Model

The bacterial and fungal strains were cultured in TSB broth. The density of microbial cells was established for 10^5^ colony forming units per mL as it was performed in MIC/MBC analyses and 100 µL of such a suspension was introduced to the wells of 96-well plate. The whole setting was incubated for 24 h/37 °C to establish the biofilm on the bottom of the well. Following this, TSB broth was removed and replaced with range of ABA concentrations in TSB analogically as it was performed in MIC/MBC analyses. Also, in the case of MBEC measurement, TTC dye was used. After colorization of survived biofilm-forming cells, the procedure of extraction of formazan was performed as described in [32]. The concentration of extracted formazan was measured using 580 nm wavelength with a spectrophotometer Multiskan Go (Thermo Fisher Scientific, Vantaa, Finland). The values of absorbance measured for biofilm treated with saline instead of ABA or PHMB comparator was considered 100%, while the biofilm reduction (%) was measured using the formula $Biofilm\;Reduction\;[\%]=100\%-\left(\frac{Abtb}{Abntb}\times 100\%\right)$, where *Abtb* is a value of absorbance measured for ABA-treated biofilm, while *Abntb* is a value of absorbance for control setting (non-treated biofilm). These tests were conducted in a total of 12 iterations in 2 timely-independent experiments.

### 4.4. Assessment of Antibiofilm Activity of ABA Antiseptics Using Biofilm-Oriented Antiseptics Test (BOAT)

The BOAT was performed following our protocol devised previously [33]. To perform the test fresh, 24 h bacterial/fungal cultures in TSB were diluted to achieve a density of 10^5^ colony forming units per mL. Such prepared suspensions were added in volume of 100 μL to wells in a 96-well plate and incubated for 24 h at 37 °C under stationary conditions to form settled biofilms. Next, the TSB was removed and 100 μL of the ABA was added to the well with adhered biofilms. The contact times were 15 min, 30 min, and 24 h, while incubation temperature was 37 °C. After exposure, the ABA was gently removed and 100 μL of the neutralizer peptone saline water (1 g/L casein, 8.5 g/L NaCl) was added for 5 min at room temperature analogically as in [21,33]. The peptone saline water was removed and 100 μL of 1% tetrazolium chloride salt (TTC, 2,3,5-triphenyl-2H-tetrazolium chloride, PanReac AppliChem, Darmstadt, Germany) in TSB, and incubated for 2 h at 37 °C. A red color indicated metabolic activity in the well and was marked as “+”; no viability (no color change) was marked as “−”. The growth control was provided by medium in well with biofilm without a test substance. The control of sterility was provided by medium with substance without a biofilm. Then, the medium was removed and 100 μL of solution of 90% methanol (Chempur, Piekary Slaskie, Poland) and 10% acetic acid was added to each well to dissolve red formazan crystals. The plate was shaken for 30 min at room temperature; afterward, the absorbance was measured at wavelength 490 nm by the MultiScan Go Spectrophotometer. The percent biofilm reduction was calculated analogically as it was performed for MIC/MBC analyses. The BOAT test was performed in 6 repeats.

### 4.5. The In Vitro Cytotoxicity Assay of ABA towards Fibroblast and Keratinocytes Cell Line

The Neutral Red (NR) cytotoxicity test was performed on fibroblasts and keratinocytes (L929 and HaCaT, ATCC, Manassas, VA, USA) These cells were exposed to ABA for 24 h. Subsequently, 150 µL of a de-staining solution (comprising 50% ethanol (96%), 49% deionized water, and 1% glacial acetic acid; sourced from POCH, Lublin, Poland) was added to wells. The 96-well plate was then shaken using MTS4 device (IKA-Labortechnik, Berlin, Germany) for 30 min. The absorbance of the NR was then spectrometrically read at a wavelength of 540 nm. The absorbance value from NR-stained cells that were not exposed to the ABA were considered 100% potential cell growth, while cells exposed to 70% EtOH (POCH, Lublin, Poland) for 30 min were used as the method’s control. These analyses were replicated six times.

### 4.6. The Bacterial Cellulose Carrier’s Synthesis, Purification and Its Soaking with ABA

The *K. xylinus* 10245, was cultivated in stationary conditions for 7 days/28 °C in a 24-well plate (VWR International, Radnor, PA, USA) in dedicated Hestrin–Schramm (H-S) medium. The BC was purified from cells/medium leftovers/impurities in 0.1 M NaOH (POCH, Lublin, Poland) for 90 min/80 °C. Next, BC carriers were immersed in distilled water and incubated while shaking. The pH value was measured every 3 h, until it reached a value of 7. The wet BC carriers of weight equal to 100 mg were transferred to the 24-well plate and immersed with 1 mL of ABA solution and left for 24 h at 8 °C. 

### 4.7. The Antimicrobial Efficacy of ABA Released from BC Carrier Measured by Modified Disk-Diffusion Method

BC carriers soaked with 1 mL of PHMB antiseptic agent or 0.9% NaCl were applied in character of control to ABA. The *S. aureus*, *P. aeruginosa,* or *C. albicans* of 0.5/0.8 McFarland density were spread over the Muller–Hinton or Sabouraud agar plates, respectively. Next, BC carriers soaked with tested compounds were placed on the plates. The whole setting was subjected to incubation at 37 °C for 24 h. After incubation, the growth inhibition zone (if occurred) was measured. The tests were performed in triplicates.

### 4.8. Manufacture of Artificial Eyeballs

The three-dimensional human eye model was prepared according to the average dimensions reported in the literature [34]. Human eye model samples were produced using laser-based powder-bed fusion of polymers (PBF-LB/P). Fuse 1 system (Formlabs Inc., Somerville, MA, USA) along with polyamide 12 powder (Nylon 12, Formlabs Inc., Somerville, MA, USA) and 110 µm layer thickness were used. The build orientation was set to ZXY.

### 4.9. The Antimicrobial Activity of ABA-BC Placed on Uneven Surface of Artificial Eyeballs Covered with Analyzed Pathogens

The artificial eyeballs were placed vertically on the M-H agar medium. The stainless steel rings were placed at 1/5 of their heights. Next, 500 µL of suspension containing 10^5^ cfu/mL of analyzed pathogens in TSB was carefully introduced to the top of the eyeball. The whole setting was prepared inside of a microbiological incubator provided with moist chamber. The pathogens were incubated for 24 h on the surface of eyeball to form adhered biofilm. Next, the ABA-BC were placed on the eyeballs for 15 min. After exposure, ABA-BC and eyeballs were placed in separate 50 mL tubes containing 5 mL of 0.1% detergent, saponine, and subjected to vortex for 1 min. The detached cells were then seeded on the appropriate agar plates using microdilution technique. The plates were then subjected for another 24 h/37 °C incubation. After 24 h, the number of grown colonies was counted and totaled (cells from eyeball and BC carrier). The setting where non-soaked BC was applied, was considered as the control of the experiment. This analysis was performed in 6 replicates.

### 4.10. Galleria mellonella In Vivo Model of Infection and Cytotoxicity Assessment In Vivo

The larvae model was used to assess ABA cytotoxicity and anti-staphylococcal activity in vivo. Sixth instar larvae of the greater wax moth, *G. mellonella*, of average weight equal to 0.21 g, were selected for the experiment. The larvae were injected with 20 µL of ABA solution (reaching 500 mg/L/larvae body mass) to evaluate its cytotoxicity or with 10 µL of the *S. aureus* 6538 strain (10^9^ CFU/mL), or with such density of *S. aureus* and 20 µL of aforementioned ABA concentration together. Moreover, a negative control with 10 µL of PBS (Biowest, Riverside, MO, USA) was used. The usability control was injection of 10 µL of 96% (*v*/*v*) ethanol (Stanlab, Lublin, Poland). The larvae were placed in 90 mm Petri dishes (Noex, Warsaw, Poland) and incubated at 30 °C/five days. Each day, the mortality of larvae was monitored. Death was defined when the larvae were nonmobile, melanized, and did not react to physical stimuli. Five groups of 10 larvae each were analyzed for each testing condition, tests were performed twice.

### 4.11. Statistical Analysis

Statistical analyses were performed using GraphPad Prism 10 (San Diego, CA, USA). Normality of distribution was verified using Shapiro–Wilk’s test. An Analysis of Variance (ANOVA) was performed to assess statistical significance. For multiple comparisons, Tukey’s post hoc test was applied. A *p*-value threshold of less than 0.05 was set for significance in the ANOVA. For the Tukey post hoc analysis, significance levels were further categorized as *p* < 0.001 and *p* < 0.0001 for specific pairwise comparisons.

## Figures and Tables

**Table 1:** Values of Minimal Inhibitory Concentration and Minimal Bactericidal (or Fungicidal) Concentration.

	MIC (µg/mL)	MBC/MFC (µg/mL)
Gram (+) *S. aureus*	64	128
Gram (−) bacteria *P. aeruginosa*	64	256
Yeast-like fungus *C. albicans*	128	512

**Table 2:** Percent reduction of microbial biofilm in particular ABA concentrations.

ABA Conc. (µg/mL)	*S. aureus*	*P. aeruginosa*	*C. albicans*
1024	77.7 ± 7.2	70.4 ± 8.3	47 ± 12.7
512	66.7 ± 14.8	39.8 ± 13.8	22.3 ± 5
256	53.3 ± 12.7	28.2 ± 10.2	17.8 ± 5.2
128	23.4 ± 7.4	0.5 ± 1.7	16.72 ± 8
64	1.2 ± 4	0.7 ± 2.6	5.5 ± 2.3
32	0 ± 3.5	0.8 ± 2.1	0.3 ± 0.8

**Table 3:** Results of quantitative biofilm oriented antiseptic test (BOAT) performed to assess activity of ABA against microbial biofilm. ABA—abietic acid, UC—utility control, PHMB—polyhexamethylene biguanide, “+”—growth; “−”—no growth.

	PHMB (UC)				ABA			
	15 min	30 min	1 h	24 h	15 min	30 min	1 h	24 h
*S. aureus*	+	+	−	−	+	**+**	**−**	−
*P. aeruginosa*	+	−	−	−	**+**	**−**	−	−
*C. albicans*	+	+	−	−	+	+	**+**	**−**

**Table 4:** The inhibition zones of microbial growth resulting from activity of 256 µg/mL of ABA released from BC.

Inhibition Zone (mm) without 15 mm Diameter of AA-BC		
*S. aureus*	*P. aeruginosa*	*C. albicans*
20.6 ± 1.15	2.3 ± 0.6	24.6 ± 0.6

## Data Availability

Data will be provided on reasonable request to the corresponding author.
